# Expressions of HLA Class II Genes in Cutaneous Melanoma Were Associated with Clinical Outcome: Bioinformatics Approaches and Systematic Analysis of Public Microarray and RNA-Seq Datasets

**DOI:** 10.3390/diagnostics9020059

**Published:** 2019-06-12

**Authors:** Yang-Yi Chen, Wei-An Chang, En-Shyh Lin, Yi-Jen Chen, Po-Lin Kuo

**Affiliations:** 1 Graduate Institute of Clinical Medicine, College of Medicine, Kaohsiung Medical University, Kaohsiung 807, Taiwan; 1050556@kmuh.org.tw (Y.-Y.C.); 960215kmuh@gmail.com (W.-A.C.); 2 Department of Dermatology, Kaohsiung Medical University Hospital, Kaohsiung 807, Taiwan; 3 Division of Pulmonary and Critical Care Medicine, Kaohsiung Medical University Hospital, Kaohsiung 807, Taiwan; 4 Department of Beauty Science, National Taichung University of Science and Technology, Taichung 403, Taiwan; eslin7620@gmail.com; 5 Department of Physical Medicine and Rehabilitation, Kaohsiung Medical University Hospital, Kaohsiung 807, Taiwan; 6 Institute of Medical Science and Technology, National Sun Yat-Sen University, Kaohsiung 804, Taiwan

**Keywords:** cutaneous melanoma, HLA class II, MHC class II, bioinformatics

## Abstract

Major histocompatibility complex (MHC) class II molecules, encoded by human leukocyte antigen (HLA) class II genes, play important roles in antigen presentation and initiation of immune responses. However, the correlation between HLA class II gene expression level and patient survival and disease progression in cutaneous melanoma is still under investigation. In the present study, we analyzed microarray and RNA-Seq data of cutaneous melanoma from The Cancer Genome Atlas (TCGA) using different bioinformatics tools. Survival analysis revealed higher expression level of HLA class II genes in cutaneous melanoma, especially *HLA-DP* and *-DR*, was significantly associated with better overall survival. Furthermore, the expressions of HLA class II genes were most closely associated with survival in cutaneous melanoma as compared with other cancer types. The expression of HLA class II co-expressed genes, which were found to associate with antigen processing, immune response, and inflammatory response, was also positively associated with overall survival in cutaneous melanoma. Therefore, the results indicated that increased HLA class II expression may contribute to enhanced anti-tumor immunity and related inflammatory response via presenting tumor antigens to the immune system. The expression pattern of HLA class II genes may serve as a prognostic biomarker and therapeutic targets in cutaneous melanoma.

## 1. Introduction

Cutaneous melanoma, a malignant tumor that originates from melanocytes, is an aggressive neoplasm with high metastatic potential that accounts for most of skin cancer related deaths. The algorithm for staging and management of malignant melanoma had been well established. However, reliable biomarkers for prediction of clinical outcome in patients with malignant melanoma are still under investigation. Since changes in protein expression patterns in melanoma may be associated with tumor progression, angiogenesis and metastasis [1,2], investigating the tumor-associated microenvironment during the early stage of tumor development may be important for predicting clinical outcome in patients with cutaneous melanoma.

Human leukocyte antigen (HLA) class II antigen expression by tumor cells was reported to influence the tumor antigen-specific immune responses and prognosis in several cancer types [3], including melanoma [4]. In humans, there are three genes *HLA-DP*, -*DQ*, and -*DR*, which encode classical major histocompatibility complex class II (MHC-II) molecules, while *HLA-DM* and -*DO* encode non-classical MHC-II molecules. Classical MHC-II proteins form a heterodimeric antigen presenting complex traditionally present in professional antigen presenting cells (APCs). *HLA-DM* molecule functions as a peptide exchange factor required for efficient loading of endosomal peptides onto MHC-II, with *HLA-DO* as its modulator. Solid tumors originating from a variety of tissues were reported to express MHC-II molecules [5]. MHC-II molecules are critical for antigen presentation to CD4+ T-lymphocytes and its role in anti-tumor immunity is increasingly appreciated. Selected tumor-associated antigens (TAA) were effectively presented by MHC-II molecules to CD4+ T cells [6,7]. Accumulating evidence also demonstrated that tumor-specific MHC-II were associated with favorable outcomes in patients with cancer [8].

Constitutively increased expression of MHC molecules in cutaneous melanoma has been reported [9,10]. A previous study also revealed that 50–60% of freshly isolated primary and metastatic melanomas express HLA class II antigens [11]. However, current available studies investigating the prognostic significance of HLA class II antigen expression in cutaneous melanoma yield conflicting results. Expression of MHC-II by tumor cells had been found positively correlated with the presence of tumor-infiltrating lymphocytes, regression of the lesion, and time to progression and overall survival of patients with metastatic melanoma [12]. In addition, it was reported to associate with a favorable outcome in cutaneous melanoma patients [13], especially in patients treated with immunotherapies [14,15,16]. In contrast, expression of MHC-II by tumor cells was also reported to associate with higher metastatic dissemination, increased tumor stage and reduced survival in melanoma [4,17]. Therefore, the functional role of MHC-II, as well as their clinical implications, remains unclear.

In the present study, to elucidate the prognostic value and functional role of MHC-II molecules in cutaneous melanoma, we systematically analyzed the correlation between transcript levels of HLA class II genes and clinical outcomes in patients with skin cutaneous melanoma based on published microarray and RNA-Seq databases.

## 2. Materials and Methods

### 2.1. Gene Expression Analysis

The expression levels of HLA class II genes in normal skin tissue and tumor samples were evaluated using the Gene Expression Profiling Interactive Analysis (GEPIA) database (http://gepia.cancer-pku.cn/) [18] which was established using microarray and RNA-Seq data from The Cancer Genome Atlas (TCGA) [19] and Genotype-Tissue Expression (GTEx) [20]. A total of 471 cutaneous melanoma patients with 480 clinical samples were included in the TCGA PanCanAtlas dataset (https://gdc.cancer.gov/about-data/publications/pancanatlas). Moreover, the expression of HLA class II genes in different sample types of cutaneous melanoma was evaluated by the UALCAN database (http://ualcan.path.uab.edu) [21]. The mRNA expression of HLA class II molecules in clinical cancer tissues and cell lines of cutaneous melanoma was analyzed by the Metabolic gEne RApid Visualizer (MERAV) [22].

### 2.2. Co-Expression Gene Analysis

To explore the HLA class II co-expressed gene, we downloaded the public microarray data of cutaneous melanoma via the web-based microarray database Oncomine (www.oncomine.org) [23] and cBioPortal [24,25]. Three cutaneous melanoma datasets were selected, including TCGA melanoma, Harlin melanoma [26] and Bogunovic melanoma [27]. Co-expressed genes with respect to *HLA-DPA1* and -*DRA* expression in cutaneous melanoma tissues were analyzed. The first dataset (TCGA provisional dataset, cutaneous melanoma) was composed of 109 primary melanoma samples and 371 metastatic melanoma samples. The second dataset (Harlin melanoma) was composed of 44 metastatic melanoma samples, 5 melanoma cell lines, and 3 normal melanocyte primary cell cultures. The third dataset (Bogunovic melanoma) was composed of 44 metastatic melanoma samples from 38 patients.

### 2.3. Survival Analysis

The Kaplan–Meier plot generation by the online databases Gene Expression Profiling Interactive Analysis (GEPIA) and UALCAN was performed to explore the association of 15 HLA class II genes and clinical outcome of cutaneous melanoma. Overall survival rate and hazard ratio estimation with respect to expression of HLA class II genes in cutaneous melanoma were also generated by GEPIA. In GEPIA database analysis, the patient samples were split into two groups (low vs. high expression). In UALCAN database analysis, the patient samples were split into three groups (low, medium and high expression). The Kaplan–Meier plot generation by UALCAN was also performed to explore the association between genes co-expressed with HLA class II genes and clinical outcome in patients affected by cutaneous melanoma.

### 2.4. OncoLnc Tool

OncoLnc (http://www.oncolnc.org) is a newly available resource for Cox coefficients and linking TCGA survival data to mRNA, miRNA or lncRNA expression [28]. Cox coefficients of HLA class II genes and their co-expressed genes were generated to explore the association of gene expressions and survival rate in cutaneous melanoma. Moreover, Cox coefficients of HLA class II genes were compared with other cancer types.

### 2.5. Gene Ontology Enrichment Analysis

Gene ontology enrichment analysis were conducted to examine *HLA-DPA1* and/or *HLA-DRA* co-expressed genes by using the Database for Annotation, Visualization and Integrated Discovery (DAVID; http://david.abcc.ncifcrf.gov/) [29]. The categories of biological process, cellular component and molecular function were selected, and all options were set as defaults. The data listed in the table were those terms with *p*-value < 0.05.

### 2.6. Statistical Analysis

The hazard ratio and p-value in survival analysis were generated by GEPIA and UALCAN. The Cox coefficient and p-value in Cox regression analysis were generated by OncoLnc. The fold enrichment and p-value in gene otology enrichment analysis were generated by DAVID. The *p*-value < 0.05 was considered statistically significant difference in all analysis.

## 3. Results

### 3.1. The Expressions of HLA Class II Genes Were Up-Regulated in Cutaneous Melanoma

**Figure 1:**
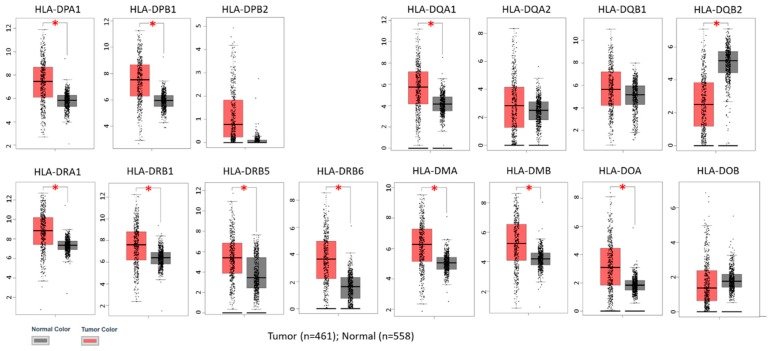
Expressions of human leukocyte antigen (HLA) class II genes in skin cutaneous melanoma from The Cancer Genome Atlas (TCGA) samples were compared with normal skin tissue from TCGA (*N* = 1) and genotype-tissue expression (GTEx) (*N* = 557) samples. Above results were obtained from the Gene Expression Profiling Interactive Analysis (GEPIA) database. The expression levels were expressed in log_2_ (TPM + 1) scale. * Indicates *p* < 0.0001 between tumor and normal tissues.

To clarify the expression pattern of HLA class II genes, the expressions of 15 HLA class II genes in skin cutaneous melanoma from TCGA samples (*N* = 461) were compared with normal skin tissue from TCGA (*N* = 1) and Genotype-Tissue Expression (GTEx) (*N* = 557) samples using the online Gene Expression Profiling Interactive Analysis (GEPIA) database. The expressions of most HLA class II genes were significantly up-regulated in cutaneous melanoma, except *HLA-DQB2*, which was significantly down-regulated in cutaneous melanoma compared to normal skin tissues (Figure 1). In addition, the expression levels of these HLA class II genes were observed to have wide range of distribution among cutaneous melanoma samples.

**Figure 2:**
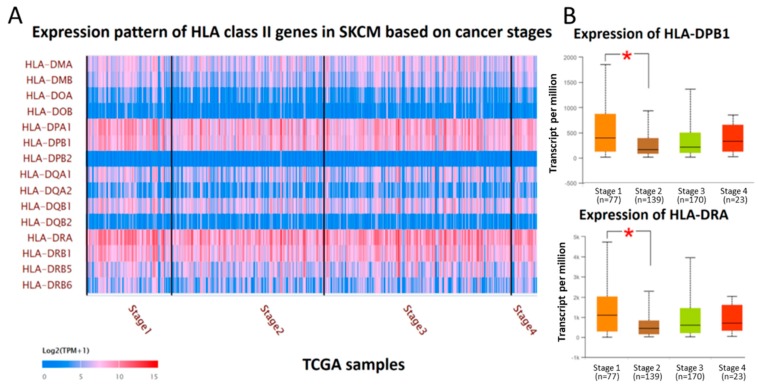
(**A**) The expression pattern of 15 HLA class II genes in each stage of cutaneous melanoma was displayed as heatmap in log_2_ (TPM + 1) scale. (**B**) The box plot represented expression of *HLA-DPB1* and *HLA-DRA* in different stages of cutaneous melanoma from TCGA samples. Significant difference (*p* < 0.05) in expressions was detected between Stage 1 and Stage 2, but not between other cancer stages.

We next investigated the changes in HLA class II mRNA expression based on cutaneous melanoma stages (Stage I, II, III, and IV according to American Joint Committee on Cancer (AJCC) TNM staging) using the UALCAN web resource. The expression pattern of 15 HLA class II genes in each stage of cutaneous melanoma was displayed as heatmap in Figure 2A. A detailed analysis of expression levels between stages was also obtained from the UALCAN database. The results indicated the expression levels of *HLA-DPB1*, -*DPB2*, -*DQA1*, -*DQB1*, - *DRA*, -*DMA* and -*DMB* were significantly higher in Stage I than in Stage II (Stage I versus Stage II, *HLA-DPB1*: *p* = 2.68 × 10^−2^, *HLA-DPB2*: *p* = 2.70 × 10^−2^, *HLA-DQA1*: *p* = 1.32 × 10^−2^, *HLA-DQB1*: *p* = 9.46 × 10^−3^, *HLA-DRA*: *p* = 5.22 × 10^−3^, *HLA-DMA*: *p* = 3.92 × 10^−2^, *HLA-DMB*: *p* = 3.23 × 10^−4^), while the expression levels of other HLA class II genes were not significantly different between Stage 1 and Stage 2. However, there was no statistical significance between other stages (*p* > 0.05), although there was a tendency of gradual increase of HLA class II gene expressions from Stage 2 to Stage 4 (Figure 2B).

### 3.2. High HLA Class II mRNA Expression Levels Were Associated with a Good Prognosis in Cutaneous Melanoma Patients

**Figure 3:**
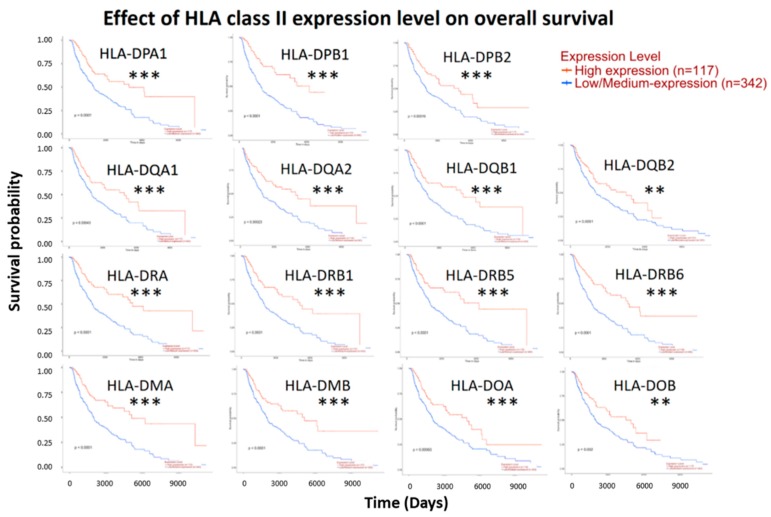
Effect of HLA class II expression level on overall survival. Survival analysis between high and low/medium expression of HLA class II genes in clinical skin cutaneous melanoma patients was performed using bioinformatics database UALCAN. Patients with high expression group had better overall survival. ** Indicates *p* value < 0.01, *** indicates *p* value < 0.001.

**Figure 4:**
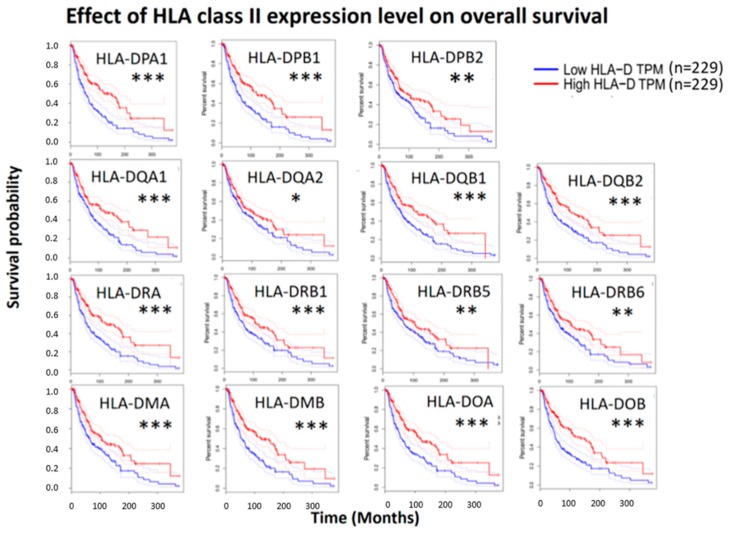
Effect of HLA class II expression level on overall survival. Survival analysis between high and low expression of HLA class II genes in clinical skin cutaneous melanoma patients was performed using bioinformatics database GEPIA. Patients with high expression group showed better overall survival. * Indicates *p* value < 0.05, ** indicates *p* value < 0.01, *** indicates *p* value < 0.001.

The survival analysis of 15 HLA class II genes in cutaneous melanoma by online databases UALCAN revealed patients with cutaneous melanoma in high expression group of all HLA class II genes had longer survival as compared with patients in low/medium expression group (Figure 3). To verify this finding, the survival analysis and hazard ratio estimation of the expression levels of 15 HLA class II genes in cutaneous melanoma were performed using online bioinformatics database GEPIA. The overall survival between high and low expression levels of 15 HLA class II genes were displayed as survival plot in Figure 4, and the relative risk of patients with high compared to low HLA class II gene expression levels was indicated in hazard ratio (Table 1). The expression fold changes between high and low expression groups were quantitated from the RNA-seq data downloaded from the cBioPortal database. Hazard ratios of <1.0 indicated patients with high HLA class II gene expressions had better overall survival.

### 3.3. The Expressions of HLA Class II Genes in Cutaneous Melanoma Were Most Closely Correlated with Patient Survival among 21 Common Cancer Types in TCGA Database

To investigate the effect of HLA class II gene expressions on survival among various cancer types, including cutaneous melanoma, the mRNA levels of HLA class II genes were examined using the bioinformatics tool OncoLnc, and the Cox regression results and the *p*-value ranks for the 15 HLA class II genes in cutaneous melanoma were listed in Table 2. A negative Cox coefficient indicated higher gene expression reduced the risk of death. Similar to results obtained from UALCAN and GEPIA databases, the expressions of HLA class II genes in cutaneous melanoma was highly correlated to patients’ overall survival. Among the 15 HLA class II genes, *HLA-DP* and -*DR* genes were found to have higher expression levels and p-value rankings in association with survival. Furthermore, the expressions of HLA class II genes in cutaneous melanoma were found more closely associated with survival in cutaneous melanoma as compared to other cancer types. As shown in Table 3, *HLA-DPA1* is the 80th gene most closely correlated with survival in cutaneous melanoma, although the expression level of *HLA-DPA1* in cutaneous melanoma was not the highest among 21 common cancer types. As shown in Table 4, HLA-DRA is the 46th most closely correlated gene in cutaneous melanoma. For both *HLA-DPA1* and *HLA-DRA*, the rank of correlations is highest in cutaneous melanoma, followed by lung adenocarcinoma, sarcoma and breast invasive carcinoma. Instead of focusing on p-values, it was suggested to focus more on the rank of the correlations, and also on the Cox coefficients in analysis by OncoLnc tool.

### 3.4. Top 11 Genes Co-Expressed with HLA-DPA1 and HLA-DRA in Clinical Cutaneous Melanoma Samples

**Figure 5:**
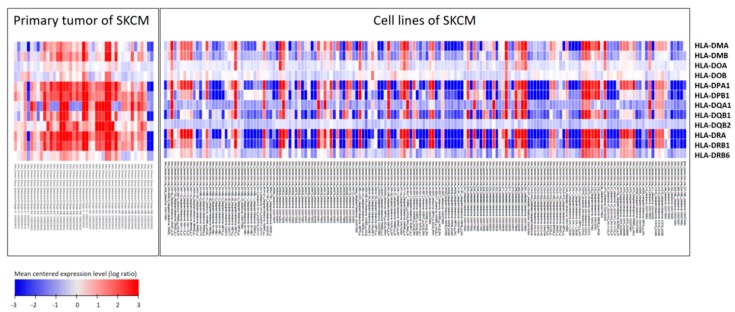
HLA class II expression pattern in cutaneous melanoma. The expression pattern of 15 HLA class II genes in primary cutaneous melanoma tumor and tumor cell lines was displayed as heatmap in log ratio of mean centered expression level. The expression pattern was observed to be more diverse in skin cutaneous melanoma cell lines than in primary tumor samples (especially expression of *HLA-DMA*, -*DMB*, -*DPA1*, -*DPB1*, -*DQA1*, -*DQB1*, -*DRA* and -*DRB1*)**.** The dataset was composed of 43 primary tumors of cutaneous melanoma and 164 cell lines of cutaneous melanoma.

**Figure 6:**
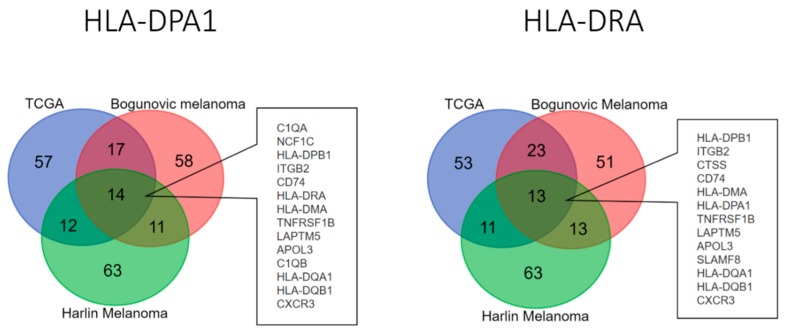
The Venn diagram analysis on the left panel showed 14 *HLA-DPA1* co-expressed genes in cutaneous melanoma from three datasets of clinical cutaneous melanoma specimens. The Venn diagram analysis on the right panel showed 13 *HLA-DRA* co-expressed genes in cutaneous melanoma from three datasets of cutaneous melanoma specimens.

The expression patterns of HLA class II genes in cutaneous melanoma from primary tumor samples and tumor cell lines were obtained from Oncomine database [23], and we found the expression patterns of HLA class II genes were different between cell lines and primary tumor samples. Specifically, as compared with in vitro cell lines of cutaneous melanoma, HLA class II gene expressions in resected tissues of melanoma tumors were generally higher, particularly *HLA-DMA*, -*DMB*, -*DPA1*, -*DPB1*, -*DQA1*, -*DQB1*, -*DRA* and -*DRB1*, as displayed in Figure 5. The phenomenon suggested that the tumor microenvironment may closely regulate the expression of these genes. Thus, we further analyzed the co-expressed genes of HLA class II genes to investigate the biological changes related to aberrant expression of HLA class II in cutaneous melanoma tissues. Using the Oncomine database and cBioPortal for public microarray data of cutaneous melanoma, we selected three datasets composed of melanoma tumor samples to analyze the genes co-expressed with HLA class II genes, specifically *HLA-DPA1* and *HLA-DRA*. In the analysis of *HLA-DPA1* co-expressed gene, 14 genes were consistently identified as top 100 genes by co-expression score in the three datasets of cutaneous melanoma; in the analysis of *HLA-DRA* co-expressed gene, 13 genes were consistently identified as top 100 genes by co-expression score in the three datasets of cutaneous melanoma (Figure 6). The genes consistently identified to be closely co-expressed to HLA class II genes were listed in Table 5, including 11 genes (*APOL3*, *CD74*, *CTSS*, *CXCR3*, *C1QA*, *C1QB*, *ITGB2*, *LAPTM5*, *NCF1C*, *SLAMF8* and *TNFRSF1B*) other than HLA class II genes.

### 3.5. Gene Ontology Enrichment Analysis of HLA-DPA1 and -DRA Co-Expressed Genes Identified the Association of These Genes with Antigen Processing, Immune and Inflammatory Response in Skin Cutaneous Melanoma

**Figure 7:**
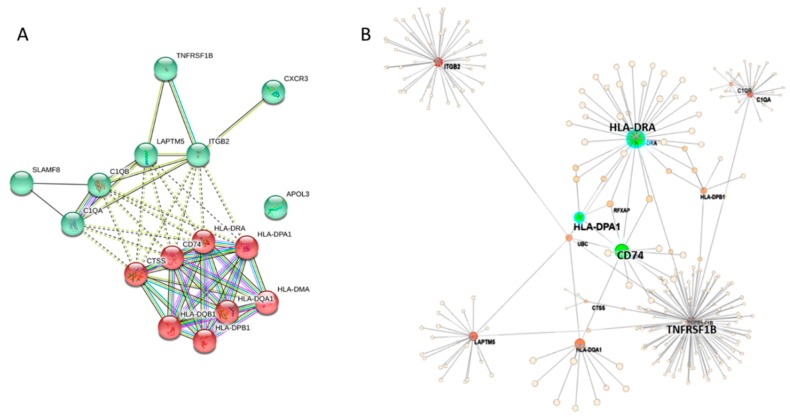
(**A**) The interaction network of co-expressed genes was generated in Search Tool for the Retrieval of Interacting Genes (STRING) database, where *CD74* and *CTSS* were clustered with HLA class II related genes. (**B**) The major interaction network of co-expressed genes generated by OmicsNet indicated *CD74* as the molecule in closest conjunction to *HLA-DPA1* and -*DRA*.

To identify the mechanisms underlying the expression of HLA class II genes and its co-expressed genes, the gene ontology enrichment analysis was conducted using Database for Annotation, Visualization and Integrated Discovery (DAVID) bioinformatics tool. Overall, 10 biological processes, 18 cellular constituents, and 4 molecular function terms were significantly enriched (*p* < 0.05, Table 6). Based on the results of the analysis, biological functions related to antigen processing and presentation and immune response were most functionally enriched, with *CTSS*, *CD74* and *TNFRSF1B* involved in these biological functions, in addition to HLA class II related genes. In addition, *CD74* was the only related gene classified in the MHC class II protein complex binding, other than HLA class II related genes. Noticeably, four genes (*APOL3*, *TNFRSF1B*, *ITGB2* and *CXCR3*) were linked to the biological process term “inflammatory response” (GO:0006954). These co-expressed genes were input into Search Tool for the Retrieval of Interacting Genes (STRING) database [30] and OmicsNet [31] for interaction network visualization. In the network produced by STRING database, CD74 and CTSS were clustered with HLA class II related genes (Figure 7A), while in the major subnetwork obtained from OmicsNet based on InnateDB database, *CD74* was the molecule in close conjunction to *HLA-DPA1* and -*DRA* (Figure 7B). Overall, the functional enrichment analysis results suggested that the cluster of genes co-expressed with *HLA-DPA1* and -*DRA* may promote anti-tumor immune response through antigen processing and presentation via MHC class II.

### 3.6. Genes Co-Expressed with HLA-DPA1 or HLA-DRA Positively Associated with Clinical Outcome in Patients with Cutaneous Melanoma

**Figure 8:**
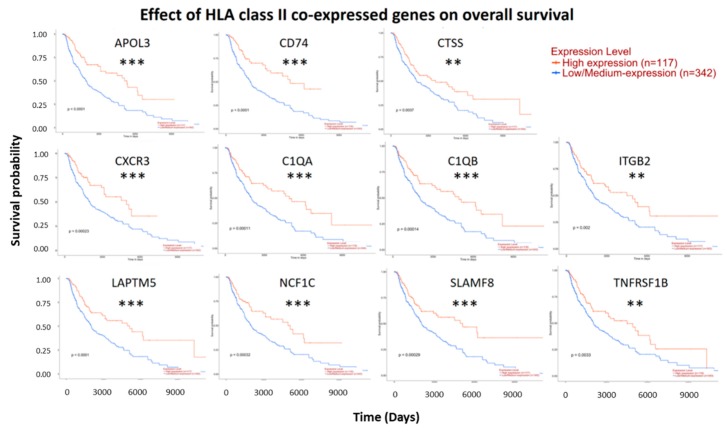
Effect of HLA class II co-expressed genes on overall survival. Survival analysis of HLA-DPA1 and/or HLA-DRA co-expressed genes in cutaneous melanoma was performed by the UALCAN database. Patients with high expression group showed better overall survival. ** Indicates *p* value < 0.01, *** indicates *p* value < 0.001.

To evaluate the significance of these 11 co-expressed genes in clinical outcome, the association of their expression levels with survival was assessed using the Kaplan–Meier plotter tool in the UALCAN web resource. The survival analysis showed higher expression level of all these 11 genes predicted a better survival in cutaneous melanoma (Figure 8). Correlation between expression level of HLA class II co-expressed genes and survival in cutaneous melanoma was also examined using the bioinformatics tool OncoLnc. Similar to the results from UALCAN database, Cox coefficients of these genes were all negative, which indicated that higher expression of these genes reduce the risk of death. *APOL3*, *CD74*, *C1QA* and *C1QB* were found to rank highest in association with survival among all co-expressed genes (Table 7).

## 4. Discussion

The present study demonstrated the novel findings that mRNA expressions of HLA class II genes, especially *HLA-DP* and -*DR*, were significantly increased in clinical cutaneous melanoma samples and positively associated with overall survival, while multivariate Cox regression analysis revealed that the HLA class II gene expressions were most closely associated with survival in cutaneous melanoma compared to other cancer types. Another novel finding in the present study is the identification of HLA class II co-expressed genes, including *APOL3, CD74, CTSS, CXCR3, C1QA, C1QB, ITGB2, LAPTM5, NCF1C, SLAMF8 and TNFRSF1B*. These co-expressed genes were associated with antigen processing and presentation, immune response and inflammatory response. The expression levels of these co-expressed genes were positively associated with overall survival in patients with cutaneous melanoma. To the best of our knowledge, this is the first study to comprehensively analyze the microarray and RNA-Seq data of HLA class II genes and their co-expressed genes in cutaneous melanoma datasets with largest sample size.

The significant association of HLA class II gene expressions with overall survival found in this study was consistent with the results obtained in large B-cell lymphoma [32], colorectal cancer [33,34,35], breast cancer [36,37], ovarian cancer [38], and laryngeal squamous cell carcinoma [39], as well as in metastatic melanoma [40]. In metastatic melanoma, positive staining for MHC-II expression in Stage III and IV cutaneous melanoma correlated with longer overall survival [40]. These studies suggested the functional role of HLA class II genes in tumor suppression. However, the available information for prognostic value of HLA class II antigens among different cancer types remained limited. In the present study, we found the rank of significance between HLA class II genes and patient overall survival was highest in cutaneous melanoma, suggesting that HLA class II genes possess greatest effect on overall survival of patients with cutaneous melanoma than other cancer types. This finding was compatible with the previous observation of special immunobiology in cutaneous melanoma [41,42]. Interestingly, cutaneous melanoma has been considered as a ‘model disease’ to investigate tumor immunobiology, to unveil the molecular basis underlying the interactions between neoplastic cells and host’s immune system, and ultimately to set up new bio-immunotherapeutic approaches [5]. However, the findings in this study were not consistent with previous study in metastatic melanoma patients, in which a low expression of HLA class II genes on neoplastic cells was associated with longer survival [43]. In that study, only 48 locoregional metastatic tumors from 39 patients were analyzed. The number of samples may be too small to draw conclusions and account for the discrepancy among these studies.

Previously, some studies focused on the change in HLA class II gene expressions during progression of cutaneous melanoma and underlying regulatory mechanisms. Here, we demonstrated that the expression levels of *HLA-DP*, -*DQ*, -*DR*, and -*DM* were significantly lower in Stage II than in Stage I cutaneous melanoma, while there was a tendency toward gradual increase in HLA class II gene expressions from Stage II to Stage IV (Figure 2). This finding was similar to the previous observation of dynamic expression of MHC during melanoma progression, and the potential mechanisms for the overexpression of MHC molecules in earlier stage were associated with the increased rates of DNA copy number gains in primary melanoma [10]. In metastatic melanoma, the master regulator of MHC genes, Class II Major Histocompatibility Complex Transactivator (*CIITA*), was significantly downregulated compared to vertical growth phase melanomas, which was considered to account for the discrepancy between expression level and DNA copy number [10,44,45].

In this study, survival analysis showed higher expression level of HLA class II co-expressed genes, especially *APOL3*, *CD74*, *C1QA* and *C1QB*, were associated with better prognosis in cutaneous melanoma. Surprisingly, *APOL3*, which encoded Apolipoprotein L3 [46] was found to be the 58th most closely correlated gene with patient survival in cutaneous melanoma, which was as high as HLA class II genes. The high expression of *APOL3* was reported to predict worse clinical outcome in patients with acute myeloid leukemia [47]. However, no study related to the functional role of *APOL3* in cutaneous melanoma has been reported. Recent studies have emphasized the association between metabolic syndrome and increased cancer risks, including skin cancer [48,49]. Non-alcoholic fatty liver disease (NAFLD), related to obesity and metabolic syndrome, has been proposed an added risk factor for extra-hepatic cancers [49,50,51], including melanoma (standardized incidence ratio of 2.4) [49]. The evidence suggests the importance of lipid metabolism in cancer development. However, whether *APOL3* directly bridge the relation between cutaneous melanoma and metabolic syndrome cannot be clarified from the results of the present study.

*CD74*, along with *CTSS* and HLA class II genes, were found involved in antigen processing and presenting via MHC-II by gene ontology enrichment analysis, while interaction network analysis identified *CD74* to be in close conjunction to *HLA-DPA1* and -*DRA*. The Cox regression analysis also indicated *CD74* as one of the higher-ranking genes positively correlated to overall survival in patients with melanoma. *CD74* is associated with development of specific adaptive immune response, and higher *CD74* expression correlated with improved recurrence-free and overall survival [52], regulated by interferon-gamma [53]. In brain metastasis, *CD74* is also a potent positive prognostic marker for survival [54]. Blockade of *CD74* related immunosuppressive signaling may restore anti-tumor immune response in metastatic melanoma, suggesting the crucial role of *CD74* in immunotherapy [55].

*C1QA* and *C1QB* encode complement C1q subunit A and B, which are the initiator of the classical complement pathway and can induce dendritic cell maturation to generate Th1 type response [56,57]. C1q appears to have pro-tumorigenic and anti-tumorigenic role in cancer, depending on the context of the disease [58]. The anti-cancer role of C1q has been reported in prostate cancer [59], as well as in syngeneic murine model of melanoma [60]. *LAPTM5* (lysosomal-associated protein transmembrane 5) is one of the top genes significantly associated with longer survival in metastatic melanoma [61], and *ITGB2* (integrin beta 2) co-expression with *HLA-DR* participates in signal transduction, cell adhesion, and motility in neoplastic melanocytes [5,62]. However, the prognostic value of *ITGB2* in cutaneous melanoma has not yet been reported. The other co-expression genes, including *TNFRSF1B*, *CXCR3*, *SLAMF8* and *NCF1*, appeared to be involved in apoptosis and inflammatory response [63,64], lymphocyte activation and T cell trafficking [65]. Taken together, the HLA class II co-expressed genes may represent the anti-tumor immune response through antigen processing as well as inflammatory response in tumor microenvironment. However, the causality of expression of HLA class II genes and their co-expressed genes in cutaneous melanoma still need to be verified in further studies.

The limitation of the current study is that the results were derived based on public cancer data repository, therefore, we validated the gene expression patterns and the results of survival analysis in the different databases available. To further investigate the prognostic value of HLA class II genes in cutaneous melanoma, collection of clinical samples with longitudinal follow up is necessary.

## 5. Conclusions

**Figure 9:**
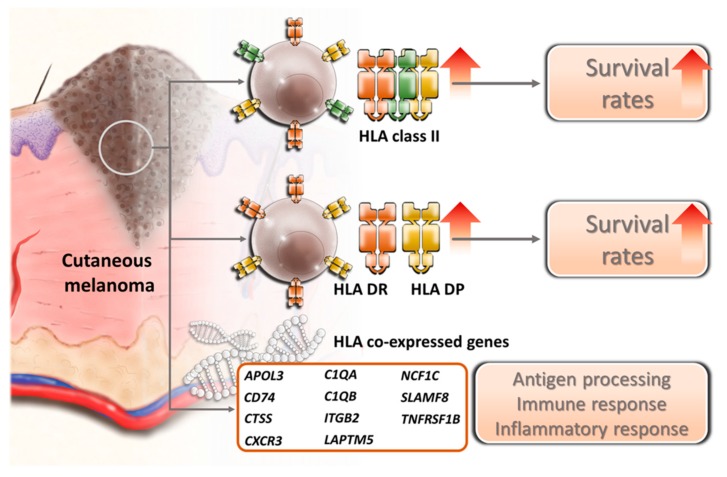
Schematic summary of the effect of HLA class II genes and their co-expressed genes in prediction of overall survival in cutaneous melanoma. The arrows indicated increased expressions and increased survival rates.

The expression levels of HLA class II in cutaneous melanoma may reflect the tumor neo-antigen presentation by tumor specific MHC-II molecules and, therefore, correlate with clinical outcome. The results of the present study indicate that the expression pattern of HLA class II and their co-expressed gene may serve as prognostic biomarkers in prediction of overall survival. A schematic summary is shown in Figure 9. Therapeutic strategies aiming to increase HLA class II expression in cutaneous melanoma may prolong overall survival, especially in combination with other immunotherapy strategies. Future studies focusing on the mechanisms involved in the regulation of expression and activity of HLA class II genes may provide further insights into the treatment of cutaneous melanoma.

## Figures and Tables

**Table 1:** Survival analysis and hazard ratio estimation of the expression of 15 HLA class II genes in cutaneous melanoma via GEPIA.

Gene	Hazard Ratio	*p*-Value	Log Rank *p*-Value	Fold Change
***HLA-DPA1***	0.50	7.1 × 10^−7^	4.3 × 10^−7^	8.01
***HLA-DPB1***	0.54	7.4 × 10^−6^	5.4 × 10^−6^	7.38
***HLA-DPB2***	0.67	0.0039	0.0037	20.28
***HLA-DQA1***	0.60	1.7 × 10^−4^	1.4 × 10^−4^	11.37
***HLA-DQA2***	0.74	0.027	0.027	13.26
***HLA-DQB1***	0.59	1.3 × 10^−4^	1.1 × 10^−4^	9.81
***HLA-DQB2***	0.56	2.0 × 10^−5^	1.5 × 10^−5^	13.51
***HLA-DRA***	0.52	1.9 × 10^−6^	1.2 × 10^−6^	8.28
***HLA-DRB1***	0.62	4.7 × 10^−4^	4.1 × 10^−4^	8.35
***HLA-DRB5***	0.70	0.009	0.0087	10.63
***HLA-DRB6***	0.65	0.0014	0.0013	11.05
***HLA-DMA***	0.62	3.9 × 10^−4^	3.5 × 10^−4^	6.06
***HLA-DMB***	0.53	4.3 × 10^−6^	3.0 × 10^−6^	6.73
***HLA-DOA***	0.54	9.2 × 10^−6^	6.8 × 10^−6^	11.39
***HLA-DOB***	0.52	2.2 × 10^−6^	1.4 × 10^−6^	17.20

**Table 2:** Association of 15 HLA class II gene expressions in cutaneous melanoma with survival generated from Cox regression analysis via OncoLnc tool.

Gene	Cox Coefficient	*p*-Value	FDR Corrected	Rank	Median Expression	Mean Expression
***HLA-DPA1***	−0.337	5.40 × 10^−7^	1.08 × 10^−4^	80	4585.31	9121.78
***HLA-DPB1***	−0.358	2.30 × 10^−7^	8.21 × 10^−5^	45	3117.18	5914.74
***HLA-DPB2***	−0.393	5.20 × 10^−8^	3.34 × 10^−5^	25	4.14	13.99
***HLA-DQA1***	−0.302	4.70 × 10^−6^	3.51 × 10^−4^	214	1170.1	2855.2
***HLA-DQA2***	−0.29	1.60 × 10^−5^	7.65 × 10^−4^	335	283.68	690.69
***HLA-DQB1***	−0.28	1.10 × 10^−5^	6.16 × 10^−4^	285	1368.12	3053.13
***HLA-DQB2***	−0.296	2.20 × 10^−5^	9.45 × 10^−4^	370	84.35	196.98
***HLA-DRA***	−0.344	2.50 × 10^−7^	8.73 × 10^−5^	46	9839.46	18,668.64
***HLA-DRB1***	−0.373	6.60 × 10^−8^	3.52 × 10^−5^	30	3544.56	7634.49
***HLA-DRB5***	−0.296	6.90 × 10^−6^	4.53 × 10^−4^	244	1079.7	2645.34
***HLA-DRB6***	−0.304	8.50 × 10^−6^	5.19 × 10^−4^	262	263.9	636.43
***HLA-DMA***	−0.335	1.90 × 10^−6^	2.01 × 10^−4^	152	1236.6	2101.88
***HLA-DMB***	−0.341	4.60 × 10^−7^	1.04 × 10^−4^	70	771.47	1417.4
***HLA-DOA***	−0.348	3.20 × 10^−7^	9.52 × 10^−5^	53	440.2	1098.24
***HLA-DOB***	−0.322	2.60 × 10^−6^	2.43 × 10^−4^	172	31.68	107.89

The Cox coefficient and *p*-value are from the gene term in precomputed multivariate Cox regressions. The false discovery rate (FDR) correction is performed per cancer analysis per data type. The rank is also performed per cancer per data type.

**Table 3:** Association of *HLA-DPA1* expression in cutaneous melanoma with survival were compared across cancer types.

Gene	Cancer	Cox Coefficient	*p*-Value	FDR Corrected	Rank	Median Expression	Mean Expression
***HLA-DPA1***	SKCM	−0.337	5.40 × 10^−7^	1.08 × 10^−4^	80	4585.31	9121.78
	LUAD	−0.235	1.50 × 10^−3^	0.031	797	9849.34	12,162.44
	SARC	−0.289	8.70 × 10^−3^	0.096	1466	4027.86	7692.16
	BRCA	−0.183	0.038	0.342	1834	5003.01	6437.27
	LGG	0.429	2.30 × 10^−5^	1.53 × 10^−4^	2513	1388.13	2947.54
	LUSC	0.106	0.120	0.636	3073	4131.19	5702.61
	CESC	−0.231	0.072	0.381	3079	3826.45	6061.82
	LAML	0.141	0.200	0.595	5017	4888.65	6455.32
	KIRC	−0.193	0.011	0.034	5394	13,119.52	15,657.27
	LIHC	−0.138	0.140	0.379	5721	2345.75	3679.56
	HNSC	−0.083	0.240	0.586	6709	3054.79	5091.7
	STAD	−0.094	0.260	0.641	6756	5108.6	7427.25
	OV	−0.068	0.380	0.851	7462	5886.04	8121.03
	UCEC	−0.069	0.510	0.995	8405	2478.44	3839.0
	BLCA	−0.072	0.320	0.585	8873	2159.67	4356.95
	GBM	0.055	0.560	0.931	10,033	5543.04	6987.86
	KIRP	−0.135	0.370	0.558	10,855	4783.41	6319.32
	READ	0.015	0.940	0.993	15,531	1829.92	2538.31
	COAD	0.0	1.00	1.00	16,386	2198.83	3337.41
	ESCA	−0.004	0.980	0.995	16,471	1974.74	3355.17
	PAAD	−0.003	0.980	0.988	17,035	5814.37	6925.07

Abbreviation: BLCA: Bladder urothelial carcinoma, BRCA: Breast invasive carcinoma, CESC: Cervical squamous cell carcinoma and endocervical adenocarcinoma, COAD: Colon adenocarcinoma, ESCA: Esophageal carcinoma, GBM: Glioblastoma multiforme, HNSC: Head and neck squamous cell carcinoma, KIRC: Kidney renal clear cell carcinoma, KIRP: Kidney renal papillary cell carcinoma, LAML: Acute myeloid leukemia, LGG: Brain lower grade glioma, LIHC: Liver hepatocellular carcinoma, LUAD: Lung adenocarcinoma, LUSC: Lung squamous cell carcinoma, OV: Ovarian serous cystadenocarcinoma, PAAD: Pancreatic adenocarcinoma, READ: Rectum adenocarcinoma, SARC: Sarcoma, SKCM: Skin cutaneous melanoma, STAD: Stomach adenocarcinoma, UCEC: Uterine corpus endometrial carcinoma.

**Table 4:** Association of *HLA-DRA* expression in cutaneous melanoma with survival were compared across cancer types.

Gene	Cancer	Cox Coefficient	*p*-Value	FDR Corrected	Rank	Median Expression	Mean Expression
***HLA-DRA***	SKCM	−0.344	2.50 × 10^−7^	8.73 × 10^−5^	46	9839.46	18,668.64
	LUAD	−0.24	1.20 × 10^−7^	0.028	706	27,710.37	34,117.21
	SARC	−0.305	5.80 × 10^−3^	0.078	1200	8466.06	16,968.25
	BRCA	−0.191	0.0292	0.313	1525	11,992.61	15,710.87
	UCEC	−0.168	0.120	0.958	1988	8596.84	11,817.06
	LGG	0.449	8.00 × 10^−6^	6.45 × 10^−5^	2084	2897.56	6102.37
	CESC	−0.242	0.064	0.362	2878	12,787.47	18,592.32
	KIRC	−0.218	3.80 × 10^−3^	0.015	4304	29,068.65	32,924.27
	LIHC	−0.156	0.100	0.316	4941	5590.88	9443.03
	HNSC	−0.095	0.180	0.520	5661	9416.73	14,015.01
	STAD	−0.107	0.210	0.590	5926	12,673.01	18,011.42
	LAML	0.116	0.270	0.659	6152	12,957.72	18,731.49
	LUSC	0.076	0.290	0.770	6291	11,713.15	18,081.96
	ESCA	−0.124	0.380	0.977	6402	5720.11	9112.51
	BLCA	−0.097	0.180	0.430	6735	5928.98	11,505.42
	OV	−0.058	0.440	0.873	8442	15,570.23	20,289.93
	COAD	−0.082	0.410	0.767	8685	6279.5	9455.2
	KIRP	−0.157	0.300	0.487	10,064	10,478.68	14,076.0
	GBM	0.036	0.690	0.958	12,029	14,344.39	19,440.02
	READ	−0.021	0.920	0.992	15,189	5247.53	7508.25
	PAAD	0.026	0.800	0.895	15,331	16,654.63	18,258.57

Abbreviation: BLCA: Bladder urothelial carcinoma, BRCA: Breast invasive carcinoma, CESC: Cervical squamous cell carcinoma and endocervical adenocarcinoma, COAD: Colon adenocarcinoma, ESCA: Esophageal carcinoma, GBM: Glioblastoma multiforme, HNSC: Head and neck squamous cell carcinoma, KIRC: Kidney renal clear cell carcinoma, KIRP: Kidney renal papillary cell carcinoma, LAML: Acute myeloid leukemia, LGG: Brain lower grade glioma, LIHC: Liver hepatocellular carcinoma, LUAD: Lung adenocarcinoma, LUSC: Lung squamous cell carcinoma, OV: Ovarian serous cystadenocarcinoma, PAAD: Pancreatic adenocarcinoma, READ: Rectum adenocarcinoma, SARC: Sarcoma, SKCM: Skin cutaneous melanoma, STAD: Stomach adenocarcinoma, UCEC: Uterine corpus endometrial carcinoma.

**Table 5:** Co-expressed genes of *HLA-DPA1* and/or *HLA-DRA* with the cut-off for selection defined as an appearance in three cutaneous melanoma datasets.

	Gene	Gene Name
Co-expressed with both ***HLA-DPA1*** and ***HLA-DRA***	*APOL3*	apolipoprotein L3
	*CD74*	CD74 molecule
	*CXCR3*	C-X-C motif chemokine receptor 3
	*HLA-DMA*	major histocompatibility complex, class II, DM alpha
	*HLA-DPB1*	major histocompatibility complex, class II, DP beta 1
	*HLA-DQA1*	major histocompatibility complex, class II, DQ alpha 1
	*HLA-DQB1*	major histocompatibility complex, class II, DQ beta 1
	*ITGB2*	integrin subunit beta 2
	*LAPTM5*	lysosomal protein transmembrane 5
	*TNFRSF1B*	TNF receptor superfamily member 1B
Co-expressed with ***HLA-DPA1***	*C1QA*	complement C1q A chain
	*C1QB*	complement C1q B chain
	*HLA-DRA*	major histocompatibility complex, class II, DR alpha
	*NCF1C*	neutrophil cytosolic factor 1C pseudogene
Co-expressed with ***HLA-DRA***	*CTSS*	cathepsin S
	*HLA-DPA1*	major histocompatibility complex, class II, DP alpha 1
	*SLAMF8*	SLAM family member 8

**Table 6:** Gene ontology (GO) enrichment analysis of HLA-DPA1 and -DRA co-expressed genes by the Database for Annotation, Visualization and Integrated Discovery (DAVID).

GO Term and Function	Count	Gene	*p*-Value	Fold Enrichment	FDR
**GOTERM_BP**					
GO:0019886, antigen processing and presentation of exogenous peptide antigen via MHC class II	8	HLA-DQB1, HLA-DPA1, CTSS, HLA-DPB1, HLA-DMA, CD74, HLA-DQA1, HLA-DRA	7.29 × 10^−13^	91.26	8.58 × 10^−10^
GO:0002504, antigen processing and presentation of peptide or polysaccharide antigen via MHC class II	6	HLA-DQB1, HLA-DPA1, HLA-DPB1, HLA-DMA, HLA-DQA1, HLA-DRA	1.66 × 10^−12^	370.41	1.96 × 10^−9^
GO:0019882, antigen processing and presentation	7	HLA-DQB1, HLA-DPA1, CTSS, HLA-DPB1, CD74, HLA-DQA1, HLA-DRA	4.56 × 10^−12^	133.5727	5.37 × 10^−9^
GO:0006955, immune response	9	HLA-DQB1, TNFRSF1B, HLA-DPA1, CTSS, HLA-DPB1, HLA-DMA, CD74, HLA-DQA1, HLA-DRA	8.06 × 10^−10^	22.44	9.49 × 10^−7^
GO:0060333, interferon-gamma-mediated signaling pathway	5	HLA-DQB1, HLA-DPA1, HLA-DPB1, HLA-DQA1, HLA-DRA	3.87 × 10^−7^	73.91	4.55 × 10^−4^
GO:0031295, T cell costimulation	5	HLA-DQB1, HLA-DPA1, HLA-DPB1, HLA-DQA1, HLA-DRA	5.66 × 10^−7^	67.28	6.66 × 10^−4^
GO:0050852, T cell receptor signaling pathway	5	HLA-DQB1, HLA-DPA1, HLA-DPB1, HLA-DQA1, HLA-DRA	7.33 × 10^−6^	35.46	0.009
GO:0006954, inflammatory response	4	APOL3, TNFRSF1B, ITGB2, CXCR3	0.004	11.08	4.88
GO:0002503, peptide antigen assembly with MHC class II protein complex	2	HLA-DMA, HLA-DRA	0.004	419.80	5.12
GO:0050870, positive regulation of T cell activation	2	HLA-DPA1, HLA-DPB1	0.016	116.61	17.26
**GOTERM_CC**					
GO:0042613, MHC class II protein complex	7	HLA-DQB1, HLA-DPA1, HLA-DPB1, HLA-DMA, CD74, HLA-DQA1, HLA-DRA	1.17 × 10^−14^	341.09	1.10 × 10^−11^
GO:0071556, integral component of lumenal side of endoplasmic reticulum membrane	6	HLA-DQB1, HLA-DPA1, HLA-DPB1, CD74, HLA-DQA1, HLA-DRA	3.06 × 10^−11^	221.79	2.88 × 10^−8^
GO:0030658, transport vesicle membrane	6	HLA-DQB1, HLA-DPA1, HLA-DPB1, CD74, HLA-DQA1, HLA-DRA	1.29 × 10^−10^	169.26	1.21 × 10^−7^
GO:0030669, clathrin-coated endocytic vesicle membrane	6	HLA-DQB1, HLA-DPA1, HLA-DPB1, CD74, HLA-DQA1, HLA-DRA	1.92 × 10^−10^	156.88	1.81 × 10^−7^
GO:0012507, ER to Golgi transport vesicle membrane	6	HLA-DQB1, HLA-DPA1, HLA-DPB1, CD74, HLA-DQA1, HLA-DRA	6.62 × 10^−10^	123.69	6.23 × 10^−7^
GO:0005765, lysosomal membrane	8	HLA-DQB1, LAPTM5, HLA-DPA1, HLA-DPB1, HLA-DMA, CD74, HLA-DQA1, HLA-DRA	1.64 × 10^−9^	31.299	1.54 × 10^−6^
GO:0030666, endocytic vesicle membrane	6	HLA-DQB1, HLA-DPA1, HLA-DPB1, CD74, HLA-DQA1, HLA-DRA	2.26 × 10^−9^	97.45	2.13 × 10^−6^
GO:0032588, trans-Golgi network membrane	6	HLA-DQB1, HLA-DPA1, HLA-DPB1, CD74, HLA-DQA1, HLA-DRA	7.28 × 10^−9^	77.49	6.85 × 10^−6^
GO:0009986, cell surface	6	HLA-DPA1, ITGB2, HLA-DPB1, HLA-DMA, CD74, HLA-DRA	7.60 × 10^−5^	11.86716	0.071
GO:0000139, Golgi membrane	6	HLA-DQB1, HLA-DPA1, HLA-DPB1, CD74, HLA-DQA1, HLA-DRA	1.14 × 10^−4^	10.88	0.108
GO:0010008, endosome membrane	4	HLA-DQB1, HLA-DPA1, HLA-DPB1, HLA-DQA1	5.23 × 10^−4^	23.178	0.491
GO:0005602, complement component C1 complex	2	C1QA, C1QB	0.002	1072	1.64
GO:0005887, integral component of plasma membrane	6	TNFRSF1B, LAPTM5, HLA-DPA1, CXCR3, HLA-DQA1, HLA-DRA	0.006	4.55	5.44
GO:0016020, membrane	7	HLA-DQB1, APOL3, ITGB2, HLA-DPB1, HLA-DMA, CD74, HLA-DQA1	0.008	3.41	7.62
GO:0005886, plasma membrane	9	HLA-DQB1, TNFRSF1B, HLA-DPA1, ITGB2, CXCR3, HLA-DPB1, CD74, HLA-DQA1, HLA-DRA	0.0149	2.34	13.19
GO:0005764, lysosome	3	LAPTM5, CTSS, HLA-DRA	0.0164	14.23	14.39
GO:0016021, integral component of membrane	10	HLA-DQB1, TNFRSF1B, HLA-DPA1, SLAMF8, CXCR3, HLA-DPB1, HLA-DMA, CD74, HLA-DQA1, HLA-DRA	0.0176	2.08	15.35
GO:0005576, extracellular region	5	C1QA, APOL3, C1QB, TNFRSF1B, CTSS	0.047	3.33	36.23
**GOTERM_MF**					
GO:0032395, MHC class II receptor activity	6	HLA-DQB1, HLA-DPA1, HLA-DPB1, HLA-DMA, HLA-DQA1, HLA-DRA	7.86 × 10^−13^	422.03	7.31 × 10^−10^
GO:0042605, peptide antigen binding	5	HLA-DQB1, HLA-DPA1, HLA-DPB1, HLA-DQA1, HLA-DRA	8.16 × 10^−9^	188.40	7.59 × 10^−6^
GO:0023026, MHC class II protein complex binding	3	HLA-DMA, CD74, HLA-DRA	8.78 × 10^−5^	197.82	0.082
GO:0004252, serine-type endopeptidase activity	3	C1QA, C1QB, CTSS	0.021	12.41	17.90

**Table 7:** Association of 11 HLA class II co-expressed genes in cutaneous melanoma with survival generated from Cox regression analysis via OncoLnc.

Gene	Cox Coefficient	*p*-Value	FDR Corrected	Rank	Median Expression	Mean Expression
***APOL3***	−0.346	3.60 × 10^−7^	9.97 × 10^−5^	58	380.28	755.47
***C1QA***	−0.346	5.30 × 10^−7^	1.08 × 10^−4^	79	2654.8	5159.04
***CD74***	−0.339	8.70 × 10^−7^	1.36 × 10^−4^	103	31,899.34	63,138.81
***C1QB***	−0.337	1.10 × 10^−6^	1.45 × 10^−4^	114	2932.11	5671.22
***ITGB2***	−0.283	3.50 × 10^−5^	1.34 × 10^−3^	418	1577.14	2848.66
***NCF1C***	−0.28	4.80 × 10^−5^	1.65 × 10^−3^	468	40.03	97.48
***CXCR3***	−0.262	5.00 × 10^−5^	1.69 × 10^−3^	474	69.42	194.29
***LAPTM5***	−0.271	5.10 × 10^−5^	1.71 × 10^−3^	478	2998.91	4868.46
***CTSS***	−0.267	7.20 × 10^−5^	2.14 × 10^−3^	537	1627.91	2518.04
***TNFRSF1B***	−0.263	1.50 × 10^−4^	3.64 × 10^−3^	661	805.72	1283.71
***SLAMF8***	−0.242	2.60 × 10^−4^	5.42 × 10^−3^	769	377.6	711.27

## Public Databases

The GEPIA [18] is an interactive web server for analyzing the RNA sequencing expression data of tumors and normal samples from the TCGA and the GTEx projects to provide differential expression analysis, survival analysis, and correlation analysis. The UALCAN [21] is an interactive web resource for analyzing cancer genomics data and these resources allow researchers to gather valuable information and data about the genes of interest. The MERAV [22] is a web-based tool for whole genome analysis which query a database comprising of about 4300 microarrays, representing human gene expression in normal tissues, cancer cell lines and primary tumors, and offers advantages in comparing gene expression among different tissue types as well as between normal and cancer cells and generating heatmaps. The Oncomine Platform [23] is a web-based microarray database that provides peer-reviewed analysis methods and contains a powerful set of analysis functions that compute gene expression signatures and automatically extracts biological insights from the data. The cBioPortal [24,25] provides visualization, analysis and download of large-scale cancer genomics datasets. The OncoLnc [28] contains survival data from patients of 21 cancer types, and is a tool for interactively exploring survival correlations and downloading clinical data coupled with mRNA expression data.
